# Shotgun metagenomic sequencing data of sunflower rhizosphere microbial community in South Africa

**DOI:** 10.1016/j.dib.2020.105831

**Published:** 2020-06-11

**Authors:** Olubukola Oluranti Babalola, Temitayo Tosin Alawiye, Carlos Rodriguez Lopez, Ayansina Segun Ayangbenro

**Affiliations:** a Food Security and Safety Niche, Faculty of Natural and Agricultural Sciences, Private Mail Bag X2046, North-West University, South Africa; b Environmental Epigenetics and Genetics Group, Department of Horticulture, College of Agriculture, Food and Environment, University of Kentucky, United States of America

**Keywords:** Illumina HiSeq, Microbiota, Metagenome, MG RAST, Soil

## Abstract

This dataset presents shotgun metagenomic sequencing of sunflower rhizosphere microbiome in Bloemhof, South Africa. Data were collected to decipher the structure and function in the sunflower microbial community. Illumina HiSeq platform using next generation sequencing of the DNA was carried out. The metagenome comprised 8,991,566 sequences totaling 1,607,022,279 bp size and 66% GC content. The metagenome was deposited into the NCBI database and can be accessed with the SRA accession number SRR10418054. An online metagenome server (MG RAST) using the subsystem database revealed bacteria had the highest taxonomical representation with 98.47%, eukaryote at 1.23%, and archaea at 0.20%. The most abundant genera were the *Conexibacter* (17%), *Nocardioides* (8%), *Streptomyces* (7%), *Geodermatophilus* (6%), *Methylobacterium* (5%), and *Burkholderia* (4%). MG-RAST assisted analysis also revealed functional annotation based on subsystem, carbohydrates sequence had 13.74%, clustering based subsystem 12.93%, amino acids and derivatives 10.30% coupled with other useful functional traits needed for plant growth and health.

Specification table

**Table:** 

Subject	Microbial Ecology, Genomics and Molecular Biology
Specific subject area	Microbial Biotechnology
Type of data	Raw metagenomics data
How the data were obtained	Bioinformatic analysis of DNA sequences performed with an online server MG-RAST
Data format	Raw data (fastq.gz.file)
Parameter for data collection	Environmental sample, Sunflower rhizospheric soil
Description of data collection	PowerSoil® isolation kit was used to extract DNA from the sunflower rhizospheric soil following the manufacture's instruction after which shotgun metagenomics sequencing was determined using Illumina HiSeq platform
Data source location	Institution: North-West University, Mafikeng, North West Province, South Africa. Samples GPS location (26.296138S: 26.972175E)
Accessibility of data	The raw sequences have been deposited in the NCBI repository with SRA accession number SRR10418054 https://www.ncbi.nlm.nih.gov/search/all/?term=SRR10418054

## 1 Value of data

• The data provides information on the rhizosphere microbiome of sunflower
• The microorganisms inhabiting the rhizosphere serves as hotspot for active biomolecules and novel genes
• Understanding the rhizosphere microbiome is important for plant growth and health
• These data are valuable, and this offers the possibilities of identifying new genes which could be an impetus for solving hunger and agricultural sustainability

## 2 Description of data

**Fig. 1:**
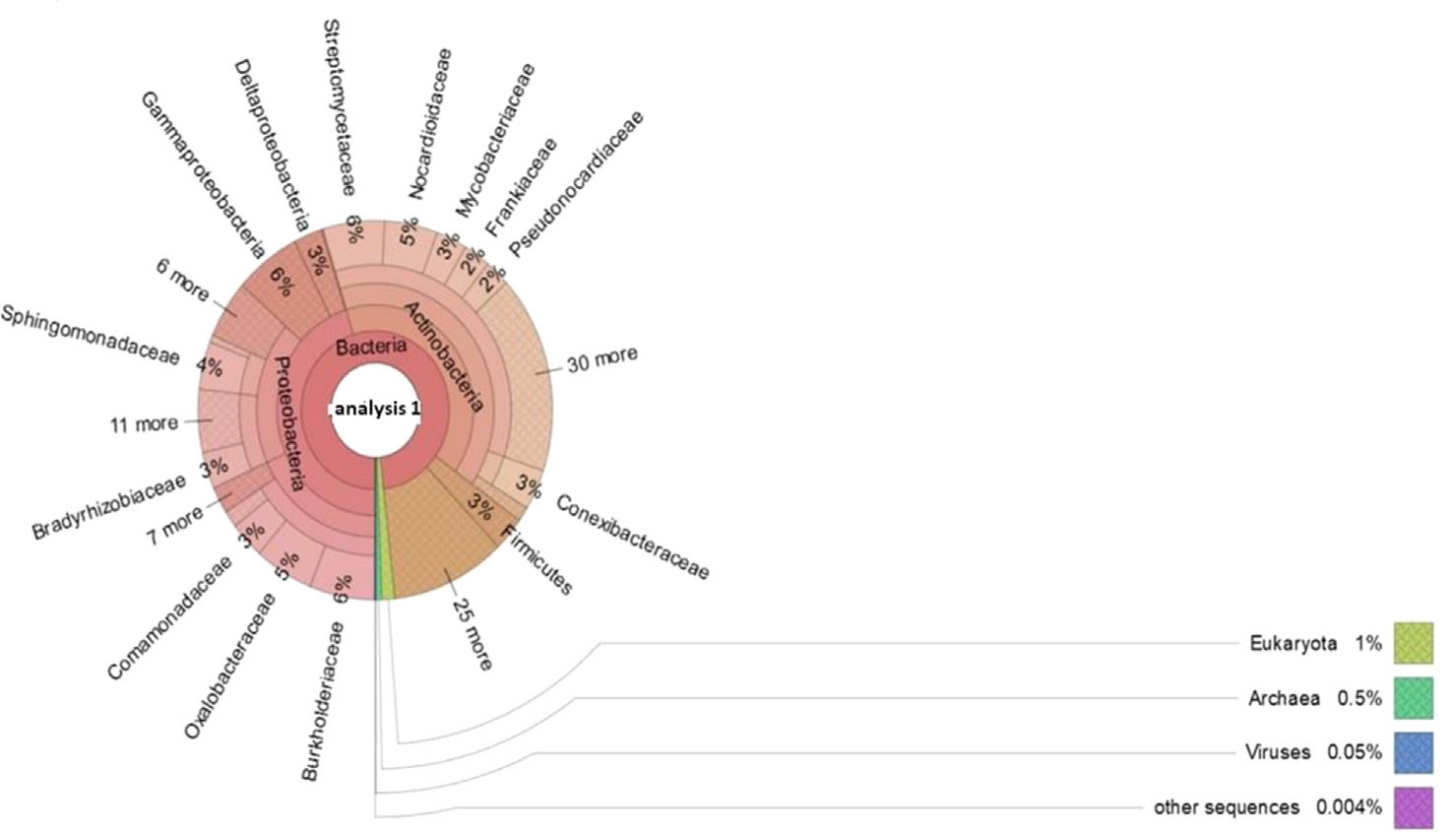
Structure of the microbiome in sunflower rhizosphere.

**Fig. 2:**
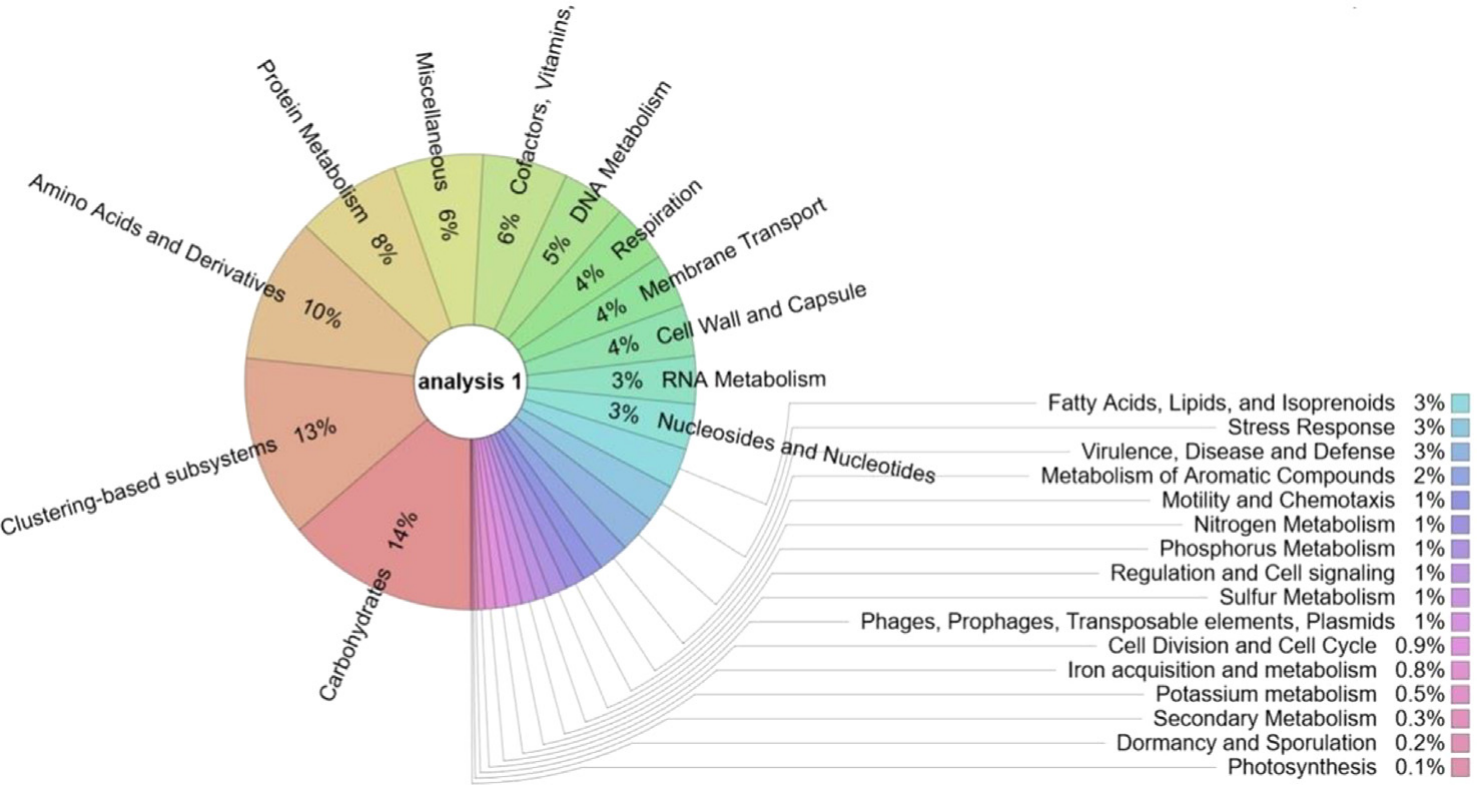
Functional structure based on subsystem in sunflower rhizosphere soil metagenome.

The dataset contains a raw sequence data obtained using shotgun metagenomic of sunflower rhizosphere microbiome. The data files in FASTQ format were deposited at the National Center for Biotechnology Information (NCBI) with SRA accession number SRR10418054. The data are presented in Fig. 1, Fig. 2, respectively.

## 3 Experimental design, materials, and methods

Soil samples were obtained from sunflower rhizosphere soils in Bloemhof, South Africa (26.296138S: 26.972175E) and the DNA samples were extracted using the PowerSoil® (MO Bio labs, USA) isolation kit following the instructions of the manufacture. DNA concentration and purity were determined using a NanoDrop Lite Spectrophotometer (Thermo Fischer Scientific, CA, USA). Extracted DNA was sent for metagenome shotgun sequencing to the Molecular Research Laboratory (www.mrdnalab.com), Texas, USA, using the Illumina HiSeq platform. Qubit® dsDNA HS Assay Kit (Life Technologies) was used to determine the initial concentration of DNA. Library preparation was done using the Nextera DNA Flex library preparation kit (Illumina) according to the manufacturer's guidelines. In brief, 50 ng of DNA were used for library preparation. After DNA fragmentation Illumina sequencing adapters were added and products amplified using six cycles of PCR during which unique indices were added. After library amplification, their concentration was estimated using the Qubit® dsDNA HS Assay Kit (Life Technologies), while the average library fragment size was measured using the Agilent 2100 Bioanalyzer (Agilent Technologies). Libraries were then pooled in equimolar ratios of 0.7 nM and sequenced paired-end for 300 cycles using the NovaSeq 6000 platform (Illumina).

The online metagenomic rapid annotation server MG-RAST (www.mg-rast.org) was used for the quality control of the raw metagenome sequences [1]. After performing quality control (QC), BLAT (the BLAST-like alignment tool) algorithm was used to annotate the sequences [2] against the M5NR database [3], which encompasses non-redundant integration of many databases.

The dataset contains 8991,566 sequences totaling 1607,022,279 bp with an average length of 179 bp. 1432,434 sequences (15.93%) failed to pass the quality control pipeline. Of those, dereplication identified 1046,824 sequences as artificial duplicate reads. Of the sequences that passed quality control, 17,387 sequences (0.24%) contain ribosomal RNA genes, 3722,027 sequences (52.74%) contain predicted proteins with known functions, and 3317,360 sequences (47.01%) contain predicted proteins with unknown function.

## Declaration of Competing Interest

The authors declare that they have no conflict of interest, either financial or commercial wise.

## Appendix Supplementary materials

Image, application 1
